# Community-informed development of an in-person forum to promote breast cancer screening and research preparedness in rural and low-socioeconomic status women

**DOI:** 10.1017/cts.2026.10788

**Published:** 2026-07-13

**Authors:** Pravesh Sharma, Kaylynn Imsande, Anni Vitriago, Maria Seibel, Joshua Pritchett, Bongi Rudder, Linh Tran, Stephanie Larson, Carolyn Flock, Crystal Murphy, Christi A. Patten, Gladys Asiedu, Folakemi Odedina, Brian A. Lynch, Tufia Haddad

**Affiliations:** 1 Department of Psychiatry and Psychology, Mayo Clinic Health System, Eau Claire, WI, USA; 2 Medical College of Wisconsin – Central Wisconsin Campus, Wausau, WI, USA; 3 Eau Claire City County Health Department, Eau Claire, WI, USA; 4 Department of Community Engagement, Mayo Clinic Health System – Chippewa Valley in Bloomer Hospital, Bloomer, WI, USA; 5 Department of Hematology/Oncology, Mayo Clinic Health System, La Crosse, WI, USA; 6 Department of Research, Mayo Clinic Health System, Eau Claire, WI, USA; 7 Department of Research Administrative Services, Mayo Clinic, Rochester, MN, USA; 8 Department of Clinical Research, Mayo Clinic Health System, Rochester, MN, USA; 9 Behavioral Health Research Program, Mayo Clinic Minnesota, Rochester, MN, USA; 10 Robert D. and Patricia E. Kern Center for the Science of Health Care Delivery, Mayo Clinic, Rochester, MN, USA; 11 Department of Hematology/Oncology, Mayo Clinic Jacksonville Campus, Jacksonville, FL, USA; 12 Department of Quantitative Health Sciences, Mayo Clinic Jacksonville Campus, Jacksonville, FL, USA; 13 Department of Pediatrics and Adolescent Medicine, Mayo Clinic, Rochester, MN, USA; 14 Department of Oncology, Mayo Clinic, Rochester, MN, USA

**Keywords:** Rural, low SES, breast cancer, WeCARE, CFIR, implementation, community engagement

## Abstract

Rural and low-socioeconomic status women face social determinants of health barriers leading to disproportionately low rates of breast cancer screening and markedly reduced participation in clinical trials. To address this gap, we developed the WeCARE (Women’s Engagement for Cancer Awareness, Resources, and Education) intervention using a community-engaged approach. This paper describes how Community Advisory Board feedback informed the development of Mayo Clinic Health System’s WeCARE intervention components. Their input was systematically analyzed using the Consolidated Framework for Implementation Research (CFIR 2.0) to identify determinants of acceptability, appropriateness, and feasibility and to guide actionable refinements to intervention content and delivery.

## Introduction

Women residing in rural areas and those with low social economic status (SES) experience substantial social determinants of health (SDoH) barriers leading to disproportionately low rates of breast cancer screening, delayed diagnoses, and poorer prognoses compared to women from urban areas and/or with higher SES [1–8]. In addition, their participation in breast cancer clinical trials remains markedly limited, reducing access to novel preventive and screening interventions [9,10].

Trialists have devoted considerable effort to improve women’s enrollment in clinical trials, primarily by recruiting those already diagnosed with cancer [11]. However, few interventions have focused on community-engaged strategies to increase both breast cancer screening and research preparedness prior to the cancer diagnosis [12]. Educating women on the clinical research process prior to disease occurrence may empower them to make informed decisions regarding their health and participation in clinical research earlier in the disease process, when there are fewer emotional and logistical barriers at play [11].

Despite these efforts, effective strategies to empower rural and low-SES women who have not engaged in or are not consistently receiving national guideline-directed breast cancer screening remain poorly understood. Although community partnerships have been widely used to support research enrollment, these efforts have largely emphasized community endorsement or downstream recruitment after diagnosis, rather than early, pre-screening engagement [11,12]. Few interventions have examined whether involving women who share similar cultural contexts, values, and lived experiences as active partners (rather than passive supporters) can build trust, increase screening uptake, and strengthen research preparedness prior to diagnosis. To address this gap, we developed the Mayo Clinic Health System (MCHS) WeCARE (Women’s Engagement for Cancer Awareness, Resources, and Education) intervention using a community-participatory approach that centers the perspectives and lived experiences of rural and low-SES women.

Emerging evidence suggests that awareness deficits alone may not be the primary driver of screening underutilization in underserved populations, and cancer centers are increasingly shifting toward implementation science frameworks that target the structural barriers disadvantaging specific populations [13,14]. WeCARE was designed in direct response to this evidence as a multi-component intervention addressing informational, cultural, and structural barriers to breast cancer screening and research participation. The intervention includes an initial community forum as the entry point, followed by three months of longitudinal navigation and support, structural barrier mitigation strategies such as coordination of local imaging services, transportation support, childcare assistance, and financial coverage through community partnerships, including connections to programs covering screening costs for uninsured and underinsured participants, and active scheduling support with local clinical providers for those with insurance.

The present paper describes the development of the community-engaged forum materials, an intentional entry point into the broader WeCARE intervention model, designed to improve knowledge, address misconceptions about clinical trials, empower women to engage with screening, and connect participants to appropriate resources based on their financial and logistical circumstances from the first point of contact.

The objectives of this research are to:

1. Characterize Community Advisory Board (CAB) feedback on the design, framing, and delivery of the WeCARE intervention, including the community forum (primary) and its supporting components.
2. Map CAB-identified themes to constructs within the Consolidated Framework for Implementation Research (CFIR 2.0) to identify key determinants of forum acceptability, appropriateness, and feasibility.
3. Document how CFIR-guided interpretation of CAB input informed actionable modifications to forum content and delivery prior to implementation, including which CAB recommendations were and were not adopted.

## Methods

### Project setting and design

This project is being conducted at the MCHS in Eau Claire, Wisconsin. Within the MCHS-Eau Claire service area, 23.0% of members live in a rural area, and 13% of the population lives below the Federal Poverty Line [15]. The project was IRB approved (IRB # 25-008934) on September 16^th^, 2025.

The WeCARE intervention concept was established through a planning process engaging local stakeholders – The Wisconsin Well Woman Program (WWWP), community oncologists, primary care physicians, the mammography team, and Mayo Clinic co-investigators. This group identified a community forum as the critical intervention entry point and established the foundational model, including the need to mitigate structural barriers and empower low-SES and rural women to engage with screening solutions. Consistent with CBPR principles, the protocol was subsequently refined with CAB feedback.

### Community advisory board

A CAB of 14 women was formed to ensure WeCARE intervention components were informed with direct input from individuals’ representative of the target population. Members were recruited from the MCHS-Eau Claire catchment area who self-identified as residing in rural and/or low-SES households, mirroring study participant inclusion criteria (*n* = 6). The remaining eight members were oncologists (*n* = 2), a faith leader (*n* = 1), a Hispanic community representative (*n* = 1), a primary care physician (*n* = 1), a practicing therapist (*n* = 1), a WWWP representative (*n* = 1), and an MCHS Community Engagement representative (*n* = 1). This composition ensured community members remained the majority voice, grounding intervention development in lived experience, while clinical and institutional members ensured clinical accuracy and systems-level feasibility. The inclusion of members reflecting both rural and low-SES backgrounds (without rigid separation) reflects the overlapping nature of these characteristics in the catchment area, where geographic isolation and economic disadvantage frequently coexist. Members were recruited through local nonprofit organizations, Mayo Clinic Connect (social network for patients and their families), and word of mouth. Prior WeCARE engagement was not required. Once membership was finalized, the principal investigator met with members to clarify roles, strengthen equitable partnership, and avoid tokenism. Compensation for CAB members was $75 for each meeting of approximately 1.5 h in length.

### Forum presentation development process

Drawing on the expertise of oncologists and breast cancer specialists within MCHS, a preliminary forum framework was developed using empirical evidence, existing literature, and collective clinical experience. CAB members then served as active co-developers of forum content and delivery through two structured virtual sessions, providing input on content sequencing, framing, terminology, language accessibility, and logistical supports. Feedback was obtained for each slide, with time allocated for comments on content, clarity, wording, flow, relevance, and delivery, as well as open-ended discussion. CAB members also shaped the post-forum follow-up model described in the results section. All decisions and revisions centered on CAB members’ lived experiences, and meetings were recorded with detailed notes documented in real time.

CAB input drove key implementation decisions, including transportation and childcare support, Spanish-language interpretation, seasonal scheduling, community-based venue selection, and mobile mammography integration that were formalized through a Mayo Clinic IRB-approved protocol amendment prior to recruitment.

### CAB feedback analysis framework

Data from CAB members were analyzed using a hybrid inductive-deductive coding approach [16]. Initial codes were developed inductively from the data to capture CAB members’ language and meanings, while deductive coding was guided by constructs within the CFIR 2.0 [17]. CFIR 2.0 provides a determinant framework for organizing stakeholder input across intervention characteristics, contextual factors, and implementation processes relevant to pre-implementation decision-making [17]. Themes were mapped to CFIR domains and constructs to characterize barriers and facilitators to forum acceptability, appropriateness, and feasibility, while remaining open to unanticipated themes from members’ lived experiences (e.g., concerns about immigration enforcement, described below). CFIR was used as an analytic lens rather than a measurement model [16–18].

## Results

CAB feedback mapped most prominently to Innovation (design and adaptability), Individuals (needs and capability), Inner Setting (culture and trust), and Implementation Process (engagement and tailoring). Outer Setting constructs were less prominent than Inner Setting and Innovation constructs, reflecting the forum’s early pre-implementation focus on content, delivery, and participant experience. However, Outer Setting – *Local Conditions* was the construct that ultimately resolved the CAB’s divergent views on racial and ethnic disparity data, underscoring that structural and equity-related barriers in the broader community shape acceptability, feasibility, and at times the resolution of competing considerations within the forum (see Table 1).

**Table 1.** Mapping Community Advisory Board (CAB) feedback to CFIR 2.0. Mapping of Community Advisory Board (CAB) feedback to constructs within the Consolidated Framework for Implementation Research (CFIR 2.0) and corresponding implementation outcomes, including acceptability, appropriateness, and feasibility, along with resulting modifications to the WeCARE forum design and delivery Table 1 long description.

CAB theme	Illustrative quote/example	CFIR domain & construct(s)	Implementation outcome(s)	Action taken/planned
Trust, representation, and credibility	“For patients, by patients”	Innovation – source, adaptability, design	Acceptability; appropriateness	Original plan to have oncologist present all content was revised to assign survivor presenters specifically to clinical trial content based on CFIR Innovation Source mapping; added CAB acknowledgment slide to reinforce community partnership and trust
Community setting and cultural alignment	Preference for trusted community location	Inner setting – culture (recipient-centeredness), relational connections, structural characteristics; individuals – need	Acceptability; feasibility	Selected rural church as forum site to enhance trust, cultural alignment, and accessibility for rural and Hispanic community members who would not attend a clinical setting
Scheduling and accessibility	Preference for mid-September timing	Inner setting – available resources; individuals – need	Feasibility	Scheduled forum in mid-September to mitigate weather-and school-related barriers; planned childcare support
Language access	Need for Spanish interpretation	Individuals – need; inner setting – access to knowledge and information	Acceptability; feasibility	Planned real-time supported Spanish translation during forum
Emotional safety and health literacy	“We are all coming from different experiences…”	Individuals – need, capability; implementation process – engaging; inner setting – culture (recipient-centered)	Acceptability; appropriateness	Considered pre-forum needs survey but rejected due to participant burden; added trigger warnings, trauma-informed language, and structured permission to take breaks within forum and pre-forum materials
Content framing and sequencing	“More calming than survival rates”	Innovation – design, adaptability	Appropriateness; acceptability	Reordered statistics (screening uptake before survival); revised terminology (e.g., “survival” → “chance of being alive years after diagnosis”)
Areas of uncertainty and divergent views	Concern about ethnic disparity framing	Innovation – design; individuals – need; outer setting – local conditions	Appropriateness	Recognized as requiring deliberation rather than immediate action. A subset of CAB members recommended removing the data; the team ultimately retained it, based on the catchment area’s changing demographics and the goal of forum adaptability to other rural communities. This was the only CAB recommendation not adopted
Research safety and physician oversight	Emphasis on protection and ethics	Innovation – evidence base, source	Acceptability; appropriateness	Revised research ethics messaging to emphasize safety, voluntariness, and physician oversight
Post-forum longitudinal support	Recommendation for ongoing contact beyond the forum	Implementation process – engaging; individuals – need; outer setting – local conditions	Feasibility; appropriateness	Designed multi-touchpoint follow-up model including phone calls at one month and three months, and mailed boosters at six weeks and two and a half months to address ongoing structural barriers including transportation, childcare, insurance navigation, and scheduling

### Trust, representation, and credibility (CFIR: Innovation – source, adaptability, and design)

CAB members emphasized that the impact of the forum depended heavily on who delivered the information. Members recommended that clinical trial–related education be presented by reputable and trusted individuals, such as breast cancer survivors, and rural residents to enhance credibility and relatability. This feedback directly changed the original intervention design, in which an oncologist had been planned to present all forum content, and reflects the Innovation – *Source* construct. The CAB feedback was itself sufficient to drive this decision, assigning breast cancer survivors to clinical trial content while retaining the oncologist for screening science. The forum shifted from a single presenter to this mixed community-clinical model based on that input. This modification reflects Innovation – *Adaptability*, and the resulting presenter model reflects Innovation – *Design.* CFIR did not validate whether the CAB feedback was correct; rather, it provided a consistent structure for documenting, categorizing, and communicating that feedback in a way that is reproducible and interpretable by other implementation science researchers and clinicians. One of the CAB members noted that the forum should be perceived as *“for patients, by patients.”*

### Community setting and cultural alignment (CFIR: Inner setting – culture; relational connections; structural characteristics; individuals – need)

CAB members emphasized that the forum setting would play a critical role in shaping participant comfort, trust, and engagement. The forum is planned to be held in a rural Lutheran church that serves as a trusted community gathering space and a major catchment site for rural residents and individuals from racial and ethnic minority groups, including Hispanic communities. CAB members indicated that a community-based setting such as a church would be more culturally aligned and approachable than a clinical or academic environment and would help reduce perceived barriers to participation. The research team had originally planned to book a standard community venue; the CAB’s feedback was itself sufficient to drive the decision to prioritize a faith-based venue instead. This reflects the Inner Setting – *Culture and Relational Connections* that helped to document that distrust of clinical and institutional settings was the underlying barrier that CAB identified. Structural characteristics of the setting, including familiarity of the physical space and accessibility for community members, were also expected to support forum delivery and participation.

CAB members recommended scheduling the forum in mid-May or September to avoid winter months and school closures that increased childcare needs. The research team had originally planned a summer date based on weather alone. The CAB’s feedback was itself sufficient to drive the decision to move the forum to mid-September. This feedback, specifically childcare and school-related constraints, reflects the Individuals – Need construct. Although childcare support is planned for some participants, CAB members indicated that mid-September and afternoon scheduling would further mitigate childcare-related barriers. In addition, CAB members suggested using real-time translation to provide live Spanish interpretation of the English-language presentation, with the goal of improving accessibility and engagement for Spanish-speaking participants. CAB members felt real-time translation of a single forum would be far superior to the option of hosting separate forums in different languages, with one CAB member stating, *“it’s a better community this way.”* This feedback mapped to Individuals – *Need*, reflecting the structural and equity-related language barriers faced by Hispanic community members in the catchment area, as well as Inner Setting – *Access to Knowledge and Information*, underscoring the need to ensure all participants could fully access forum content regardless of language.

### Emotional safety and health literacy [CFIR: Individuals – need and capability; implementation process – engaging; inner setting – culture (recipient-centered)]

CAB members highlighted the emotional vulnerability of individuals engaging with breast cancer information and emphasized the importance of trauma-informed delivery strategies to support emotional safety and comprehension. Feedback reflected variation in participants’ emotional readiness and health literacy, underscoring the need to address individual-level needs and capabilities. This feedback maps to the Individuals – *Need and Capability* construct. This insight prompted consideration of a brief pre-forum needs survey to assess participant readiness in advance; however, following deliberation, the team determined that administering a survey prior to the forum would impose unnecessary burden on participants who were already being asked to complete baseline assessments. The team therefore prioritized integrating proactive supports directly into the forum itself, including trigger warnings at the beginning of the forum and within pre-forum materials, explicit normalization of emotional responses, and structured permission for participants to take breaks as needed. One CAB member summarized this approach by noting, *“We are all coming from different experiences. Feel free to step out if you need to take a break and rejoin when you feel ready.”*

### Content framing and sequencing (CFIR: Innovation – design; adaptability)

CAB members supported inclusion of breast cancer screening and survival data but emphasized the importance of framing and sequencing the slides to support emotional readiness. Members recommended presenting less emotionally distressing statistics, such as screening uptake data (rural vs. urban), prior to survival statistics, which were described as *“more calming”* and better suited for early engagement. CAB members also advised revising terminology to improve clarity and reduce distress; for example, reframing *“5-year survival”* as *“chance of being alive 5 years after diagnosis.”*

### Areas of uncertainty and divergent views (CFIR: Innovation – design; individuals – need; outer setting – local conditions)

Although CAB members broadly agreed on the importance of trauma-informed framing, some expressed uncertainty about whether racial and ethnic survival disparities should be presented without additional contextual explanation, with one CAB member asking, *“Why do differences in survival rates between ethnicities matter?”* A subset of members raised concerns that inclusion of these disparities, if not carefully framed, could shift focus away from the forum’s primary emphasis on rural vs. urban resident and SES disparities and increase emotional burden for participants. Some members also felt that, because the Midwest catchment population is predominantly White, this data was less locally relevant and could distract from the forum’s focus on women as a whole. Mapping this feedback to Innovation – *Design* and Individuals – *Need* exhibited a tension between intervention scope and participant emotional readiness that required deliberation rather than immediate action. Resolving this tension required looking beyond the forum itself to the Outer Setting – *Local Conditions* construct, which asks what is happening in the broader community outside the forum: who lives there and what do they need. This reflected the growing diverse population within the rural catchment area [15] and the team’s goal of making the forum adaptable to other rural communities. On this basis, the research team ultimately decided to keep the racial and ethnic disparity data despite the feedback to remove it. The team shared this reasoning with the CAB to maintain transparency and trust. This was the one instance where CAB feedback was not adopted; CFIR helped the team work through the competing considerations and explain its decision clearly.

### Research safety and physician oversight (CFIR: Innovation – evidence base; source)

CAB members emphasized the importance of clearly communicating the safety of clinical research and the role of physician oversight in protecting participants. Members also stressed the importance of utilizing simple language, such as *“protecting you”* or *“doing the right thing,”* instead of more complex terms, such as *“ethics in research.”* This feedback mapped to Innovation – *Evidence Base*, reflecting the need to reinforce perceptions of research legitimacy, ethical conduct, and participant protection, and to Innovation – Source, given the role of trusted clinicians and institutions in conveying safety. Based on this feedback, the team revised research ethics messaging throughout the forum to emphasize safety, voluntariness, and physician oversight using the simpler language the CAB recommended.

### Post-forum longitudinal support and structural barrier mitigation (CFIR: Implementation process – engaging; individuals – need; outer setting – local conditions)

CAB members emphasized that a single educational forum would be insufficient to support sustained screening engagement among rural and low-SES women facing ongoing structural barriers. Members recommended a multi-touchpoint longitudinal approach to maintain connection with participants beyond the forum and continue actively supporting mitigation of structural barriers to screening. This feedback mapped to Implementation Process – *Engaging*, reflecting the need for sustained participant engagement beyond a single implementation event, as well as Individuals – *Need*, given the ongoing and variable nature of barriers such as transportation, childcare, insurance navigation, and scheduling that participants would continue to face after the forum. Outer Setting – *Local Conditions* was also critical here, underscoring that structural barriers in the external community context required active and ongoing navigation rather than one-time informational support.

Based on this input, the post-forum follow-up model was designed to include a structured phone call at one month (T2) and three months (T3), a culturally tailored mailed booster at approximately six weeks, and a second mailed booster at approximately two and a half months. Each touchpoint was designed to reinforce screening engagement, address barriers such as transportation, childcare, insurance navigation, and scheduling, and sustain connection between participants and local clinical providers. This CAB guided follow-up model extends the WeCARE intervention’s focus on structural barriers beyond the forum itself, reflecting the CAB’s input that education alone cannot overcome the structural barriers this population faces.

### Application of CAB feedback

Based on CAB input, the research team revised slide sequencing, simplified survival terminology, added trigger warnings, clarified screening pathways, and revised research ethics messaging to emphasize safety, voluntariness, and participant protection. Overall, academic and clinical authority were intentionally balanced with community expertise by treating CAB members as co-partners in decision-making, prioritizing lived experience alongside technical knowledge, and incorporating community recommendations even when they challenged initial researcher assumptions. In one instance, it meant the research team’s broader implementation goals took precedence over a specific CAB recommendation, a decision that was shared transparently with the CAB.

## Discussion

This research fulfilled its objectives by systematically characterizing CAB feedback, mapping CAB-identified themes to relevant CFIR 2.0 domains and constructs to identify key determinants of forum acceptability, appropriateness, and feasibility, and translating these insights into actionable modifications to forum content and delivery prior to implementation. Across multiple instances, CAB feedback was itself sufficient to drive implementation decisions. CFIR’s contribution was the documentation and communication of those decisions within a validated implementation science framework, ensuring they are reproducible and interpretable by implementation science researchers and clinicians. One CAB recommendation, to remove racial and ethnic disparity data from the forum, was not adopted. This was the only instance in which CAB feedback was not acted upon. Resolving it required moving beyond the constructs that defined the disagreement (Innovation – *Design* and Individuals – *Need*) to a third construct, Outer Setting – *Local Conditions*, which pointed to the catchment area’s changing demographics and the team’s goal of making the forum adaptable to other rural communities. This shows that CFIR mapping can do more than document consensus; it can also surface considerations that justify departing from CAB input, provided that the decision is communicated back to the CAB transparently.

The ongoing nature of our community engagement was demonstrated when, following completion of the formal second CAB session, a CAB member proactively raised concerns about fear of immigration enforcement as an emerging barrier to participation among Spanish-speaking community members. This reflects the strength and continuity of the community partnership, demonstrating that the CAB model fostered trust and open communication that extended beyond formal data collection windows and remained responsive to evolving community needs.

Our approach has limitations. While this project was designed with CBPR principles and involved multi-stakeholder co-design of the intervention model, the community forum format was established prior to the formal CAB content development process, and future iterations should build in earlier community input on intervention format decisions to more fully grasp CBPR co-design principles. Additionally, CFIR was applied only after CAB feedback was collected, as a deductive lens for organizing and interpreting that feedback, rather than a priori to structure CAB discussion guides; future work could incorporate the CFIR interview guide tool prospectively to help identify community input gaps across these domains. Relatedly, only one CAB recommendation was not adopted in this project; this single instance is informative but insufficient to establish how reliably CFIR mapping can help resolve disagreement. Future research involving similar situations may help test this further. Findings may not fully generalize to populations without prior exposure to research partnerships and/or populations from differing rural areas.

This work may be most useful to other CBPR researchers who collect substantive CAB feedback but lack a structured method for organizing it within implementation science. CBPR aims not only to honor community expertise but also to advance translation into real-world practice, and that translational goal requires documentation that implementation science audiences can interpret and build on. Mapping CAB feedback to CFIR offers one way to meet both goals at once. It preserves CAB members as essential co-partners in shaping intervention design while giving researchers a structured, reproducible way to communicate why those decisions matter, to whom, and under what conditions they might generalize to other settings.

## Table 1. Long description

The table has 10 rows and 5 columns. The columns are labeled CAB theme, Illustrative quote/example, CFIR domain & construct(s), Implementation outcome(s), and Action taken/planned. The rows detail various themes such as Trust, representation, and credibility; Community setting and cultural alignment; Scheduling and accessibility; Language access; Emotional safety and health literacy; Content framing and sequencing; Areas of uncertainty and divergent views; Research safety and physician oversight; and Post-forum longitudinal support. Each row includes specific illustrative quotes or examples, corresponding CFIR domains and constructs, implementation outcomes, and actions taken or planned.

Navigate back to Table 1..
